# Ultrasound-Guided Regional Anesthesia for Procedures of the Upper Extremity

**DOI:** 10.1155/2011/579824

**Published:** 2011-05-30

**Authors:** Farheen Mirza, Anthony R. Brown

**Affiliations:** College of Physicians and Surgeons, Columbia University, New York, NY 10027, USA

## Abstract

Anesthesia options for upper extremity surgery include general and regional anesthesia. Brachial plexus blockade has several advantages including decreased hemodynamic instability, avoidance of airway instrumentation, and intra-, as well as post-operative analgesia. Prior to the availability of ultrasound the risks of complications and failure of regional anesthesia made general anesthesia a more desirable option for anesthesiologists inexperienced in the practice of regional anesthesia. Ultrasonography has revolutionized the practice of regional anesthesia. By visualizing needle entry throughout the procedure, the relationship between the anatomical structures and the needle can reduce the incidence of complications. In addition, direct visualization of the spread of local anesthesia around the nerves provides instant feedback regarding the likely success of the block. This review article outlines how ultrasound has improved the safety and success of brachial plexus blocks. The advantages that ultrasound guidance provides are only as good as the experience of the anesthesiologist performing the block. For example, in experienced hands, with real time needle visualization, a supraclavicular brachial plexus block has changed from an approach with the highest risk of pneumothorax to a block with minimal risks making it the ideal choice for most upper extremity surgeries.

## 1. General Anesthesia versus Regional Anesthesia

Anesthesia options for upper extremity surgery include general anesthesia, regional anesthesia, or a combination of the two. In the past general anesthesia was frequently the method of choice for upper extremity surgery due to lack of training and experience with regional anesthesia as well as fear of complications including vascular puncture, local anesthetic toxicity, pneumothorax, and patient discomfort [1]. Needle placement utilizing the paresthesia technique or peripheral nerve stimulator could be a time-consuming process leading to operating room delays and patient discomfort. However, the advantages of general anesthesia, including control of the airway and familiarity of the technique by the majority of anesthesiologists are overshadowed by the clear benefits of regional anesthesia. These include intraoperative, as well as postoperative analgesia [2, 3]. In addition, regional anesthesia results in excellent muscle relaxation during surgery, decreased opioid requirements and their potential side effects, greater hemodynamic stability, increased efficiency in the operating room by avoiding the time required to awaken and extubate the patient, reduced PACU stay, a decrease in unplanned hospital admission for pain control, as well as greater patient satisfaction [2, 3]. The most significant advantage of regional anesthesia for surgery of the upper extremity is the prolonged postoperative analgesia that a nerve block can provide. The pain relief following brachial plexus blockade with long-acting local anesthetics such as bupivacaine, ropivacaine and levobupivacaine has resulted in patients being discharged home on the day of surgery as opposed to a planned or unplanned overnight admission [3, 4].

## 2. Brachial Plexus Anatomy

Thompson and Rorie performed cadaveric studies to map out the brachial plexus anatomy [5]. The fifth through eighth cervical and first thoracic nerve exit through the intervertebral foramina and travel along the groove formed by the transverse processes of their corresponding vertebrae. After exiting the transverse processes the roots of the brachial plexus travel between the anterior and the middle scalene muscles, identified as the interscalene groove [1]. The authors reported that the brachial plexus is confined by a continuous fascial sheath formed by the deep cervical fascia and that the fascia is continuous from emergence of the nerve roots to the axilla. Distally the fascia folds inwards to form separate compartments for each nerve. For example, at the cord level, local anesthetic injected around the posterior cord may not spread to include the lateral or medial cords [6–8] whereas an axillary brachial plexus block is performed by identifying the individual nerves and blocking each one separately [9–11]. While an interscalene block is performed by means of a single injection technique, the more distal approach to the brachial plexus, the less likely that a single injection technique will result in complete blockade of the upper extremity [12–14].

## 3. Basics of Ultrasound

Ultrasound probes (transducers) act as both a transmitter and receiver of sound waves. The probes are classified as either high (10–15 MHz), midrange (5–10 MHz), or low (<5 MHz) frequency. High-frequency probes provide high-resolution images but lack depth of penetration compared to low-frequency probes [15]. Both frequency types are available with a wide or a narrow footprint. High-resolution linear transducers are most suitable for imaging superficial structures such as the brachial plexus in the interscalene, supraclavicular, and axillary regions. The lower frequency curved transducer is preferable when the anatomical structures are deeper than 4 cm, for example, when performing an infraclavicular block [16]. Prior to the use of ultrasound, block needle placement was achieved using a blind approach with the nerve stimulation or paresthesia technique. Ultrasound imaging has revolutionized the practice of regional anesthesia in that the operator can visualize and identify nerves and blood vessels as well as the needle during its passage through the tissues. Abnormal anatomy can also be recognized [17]. In addition, direct visualization of the spread of local anesthetic decreases the risk of intravascular injection, local anesthetic toxicity, pneumothorax, and a failed block [18]. It is important to remember, however, that the success of an ultrasound-guided block is dependent upon the skill and experience of the anesthesiologist. Anesthesiologists performing brachial plexus anesthesia under ultrasound guidance must first become comfortable with identifying anatomical structures as well as visualizing the needle during the block performance. In experienced hands, the benefits of performing a peripheral nerve block with real-time ultrasound imaging of needle placement and local anesthesia spread include decreased performance as well as onset time, a decreased dose of local anesthetic required to achieve a successful block, and an increase in block success rate [19–24]. In a systematic review and meta-analysis of randomized controlled trials comparing ultrasound guidance with electrical neurostimulation for peripheral nerve blocks, Abrahams et al. confirmed these aforementioned benefits of ultrasound. However, the authors concluded that larger studies are needed to determine whether ultrasound can decrease the number of complications [25].

## 4. Patient Preoperative Evaluation and Education

The success of a regional anesthesia program is dependent on patient education and the support of the surgical team. To put a patient at ease it is desirable for the surgeon to inform the patient about the possibility of receiving a brachial plexus block for his or her surgery prior to the day of surgery. A patient that has been informed beforehand is often more amenable to accepting regional anesthesia. In addition, the training, education, and skill of the individual performing the block are of paramount importance. To this end, both the American as well as the European Society of Regional Anesthesia (ASRA and ESRA) hold annual meetings as well as numerous workshops throughout the year to educate and train individuals in the art of regional anesthesia [26]. There are few absolute contraindications to a brachial plexus block. These include patient refusal, local anesthetic allergy, infection at the site of needle entry, and the presence of infected lymph nodes in the axilla or supraclavicular region [7, 27]. Deep blocks, for example, an infraclavicular approach, as well as blocks in the vicinity of a noncompressible artery (e.g., supraclavicular) should not be performed in coagulopathic patients [6, 7]. A patient that is unable to cooperate secondary to decreased mental status is also an absolute contraindication. Regional anesthesia is not contraindicated in patients that have a pre-existing stable neurological deficit or chronic neurological disease provided that the condition is well documented [28, 29]. It is up to the anesthesiologist to decide, based on each individual patient's risk benefit ratio whether performance of a peripheral nerve block in the presence of pre-existing nerve damage is indicated [30]. An informed cooperative patient is an essential factor in ensuring safe and effective regional anesthesia. Following a brachial plexus block, it is essential that the affected extremity be immobilized and protected until loss of sensation and proprioception have resolved. Patients and family members should receive clear instructions regarding the anticipated duration of the block and how to transition to oral analgesia at home to avoid the sudden onset of pain.

## 5. Anesthetic Techniques

De Andres and Sala-Blanch state that it is essential to understand both the topographic anatomy and cross-sectional anatomy of each anatomic zone of the brachial plexus [15]. They describe the brachial plexus as being divided into three zones: the supraclavicular region in the posterior triangle of the neck, the infraclavicular region deep to the pectoralis muscles in the anterior chest, and the axillary region. The level of needle entry in one of these zones will determine the extent, limitations, and potential complications of a brachial plexus block. Prior to the use of ultrasound, the likelihood of encountering the spinal cord, the lung, and major vessels such as the subclavian and vertebral arteries with the more proximal approaches (interscalene and supraclavicular) was a concern [31]. Ultrasound has minimized these risks provided that the needle tip as well as the spread of local anesthetic is constantly visualized throughout performance of the block [20, 32]. The choice of which technique to use is dependent on the surgical procedure, the comfort and expertise of the anesthesiologist, and patient-associated factors such as sepsis in the axilla. In the latter case a more proximal approach is desirable [30].

## 6. Interscalene Block

The interscalene approach to the brachial plexus is the technique of choice for surgical procedures of the shoulder. It is inappropriate for surgeries involving the medial aspect of the upper extremity due to inconsistent blockade of the lower trunk (C8 and T1) [33, 34]. At the level of the cricoid cartilage the brachial plexus trunks appear as three distinct hypoechoic areas between the anterior and middle scalene muscles [1, 35]. It should be emphasized that the large doses of local anesthetic traditionally used for an interscalene block with the neurostimulation technique result in a 100% incidence of ipsilateral phrenic nerve paralysis due to blockade of the 3rd, 4th, and 5th cervical nerve roots. This may decrease the patient's FRC by 25% [36] and may therefore not be suitable for patients with emphysema and other chronic lung diseases with decreased pulmonary reserve. Ultrasound imaging improves the interscalene approach primarily by being able to visualize the spread of local anesthetic within the fascia surrounding the trunks. This direct visualization decreases the amount of local anesthetic needed to provide surgical anesthesia [37, 38]. Decreasing the volume of local anesthetic to 10 mL or 5 mL resulted in a significant decrease in the incidence of hemidiaphragmatic paresis [37, 39, 40]. Kapral et al. report that ultrasound guidance improves both the quality of the nerve block and shortens the time of onset of sensory blockade [22].

## 7. Supraclavicular Block

The supraclavicular block was traditionally performed for surgeries of the upper extremity below the shoulder. Liu et al., however, recently reported that ultrasound-guided supraclavicular blocks are effective and safe for shoulder arthroscopy [41]. The supraclavicular approach has several advantages over the more distal approaches including rapid onset of the block, more complete blockade of the nerves supplying the upper extremity (with the exception of the intercostobrachial nerve) due to the compact arrangement of the trunks of the brachial plexus at this level [32, 42]. Prior to the use of ultrasound the supraclavicular approach was frequently avoided, particularly in ambulatory surgeries, due to the increased risk of pneumothorax and, to a lesser extent, direct vascular puncture of the subclavian, superficial (transverse) cervical, suprascapular, or dorsal scapular arteries with subsequent local anesthetic toxicity and cardiovascular collapse [43, 44]. Ultrasound has improved the safety of a supraclavicular block as the anesthesiologist can now visualize the subclavian artery, the first rib, as well as the dome of the lung. Placement of the needle and spread of the local anesthetic can now be seen in real-time resulting in resurgence in the use of this block [17, 45]. Chan et al. examined the supraclavicular region in 40 patients and reported that in all cases the nerves of the brachial plexus appeared as hypoechoic nodules in clusters lateral, posterior, and cephalad to the subclavian artery [46]. The authors also concluded that if the needle is seen at all times and not inserted beyond the first rib, then the risk of a pneumothorax in a supraclavicular block is essentially eliminated. Williams found that supraclavicular nerve blocks were performed faster with ultrasound guidance when compared with nerve stimulation (5 versus 10 min) [20]. Ultrasound guidance has increased the safety profile of the supraclavicular approach so that in experienced hands this may be the block of choice for most upper extremity surgeries below the shoulder.

## 8. Infraclavicular Block

The infraclavicular approach is indicated for surgeries of the arm and hand. Compared to the supraclavicular approach, the risk of pneumothorax is significantly reduced and is virtually eliminated with the use of ultrasound. In addition, the phrenic nerve is not blocked with this approach [47]. Compared to the axillary approach, the infraclavicular block targets the brachial plexus at the level of the cords which surround the second part of the axillary artery and are proximal to the takeoff of the musculocutaneous, axillary, and medial brachial cutaneous nerves. This may result in a higher success rate of complete blockade with a single injection technique [48]. The infraclavicular anatomy may, however, be more difficult to visualize under ultrasound guidance particularly in obese patients. Perlas et al. found that compared to the interscalene, supraclavicular, axillary, and midhumeral approaches in which the brachial plexus was visualized 100% of the time, in only 27% of patients were they able to visualize the infraclavicular brachial plexus [1, 49]. This difficulty is due to the relative depth of the brachial plexus in the infraclavicular approach compared to all other approaches to the brachial plexus. A low-frequency probe with its greater tissue penetration may facilitate performance of this block [47]. In an ultrasound-guided infraclavicular block the lateral, posterior, and medial cords are seen in close proximity to the axillary artery and vein. The posterior cord is usually blocked first. If the spread of local anesthetic does not surround the lateral and medial cords, then all three cords must be blocked individually to obtain complete blockade of the upper extremity. As with the supraclavicular and axillary approaches, the intercostobrachial nerve will have to be blocked separately in the axilla to achieve anesthesia of the inner aspect of the upper arm.

## 9. Axillary Block

Axillary blocks are performed for procedures of the elbow, distal arm, and hand. Prior to the use of ultrasound, the axillary approach was the most common approach to the brachial plexus due to the safety of this technique. As with the infraclavicular block, the risk of phrenic nerve paresis is avoided and the risk of pneumothorax is eliminated. The high success rate without respiratory compromise makes this block desirable in patients with reduced lung capacities and chronic pulmonary diseases [50]. Contraindications to the axillary approach include inability to abduct the arm to the position necessary to perform the block, localized infection in the axilla, or enlarged axillary lymph nodes [51]. A major advantage of using ultrasound in an axillary approach is the ability to confirm blockade of the musculocutaneous nerve [52]. Because of the anatomical variance of the musculocutaneous nerve in relation to the axillary artery, failure to block this nerve with a perivascular approach in not uncommon [53]. At the axillary level, the terminal branches of the brachial plexus (median, ulnar, and radial nerves) are situated close to the axillary artery and veins with the two axillary veins situated medial to the artery. There is, however, a great deal of variation in the distribution of these three nerves in relation to the artery [54]. The four nerves are easily visualized utilizing ultrasonography. The ultrasound guided axillary approach has been shown to both decrease block failure rate and time of onset of sensory blockade compared to the transarterial technique [14]. The success of US guided axillary blocks depends on the multiple needle approach in which each nerve is identified individually and spread of local anesthetic is observed around the median, ulnar, radial, and musculocutaneous nerves.

## 10. Distal Upper Extremity Blocks

Individual terminal nerve blocks can be performed at the midhumeral, elbow, forearm, or wrist either by design or as a rescue block [7]. These more peripheral nerve blocks may be performed to achieve postoperative analgesia while at the same time maintaining more proximal control of the upper extremity.

## 11. Postoperative Pain Control

Postoperative pain and nausea are the leading causes of unplanned hospital admission after ambulatory surgery [55]. Orthopedic upper extremity surgery is reported as having a high incidence of severe pain [56]. One of the clear benefits of regional anesthesia over general anesthesia for upper extremity surgery is the postoperative pain relief a long-acting local anesthetic can provide. The choice of local anesthetic is determined by the duration of surgery, necessity of motor blockade, urgency of neurological assessment after surgery, and the anticipated requirement for postoperative analgesia. In brachial plexus nerve blocks short-, intermediate-, and long-acting local anesthetics can be chosen. Bupivacaine, ropivacaine, and levobupivacaine are equally effective in surgeries in which extended postoperative analgesia would be beneficial. In comparison to bupivacaine, however, ropivacaine and levobupivacaine are the long-acting local anesthetics of choice due to their decreased cardiotoxicity [57–59]. It is important for the patient to be informed of the anticipated duration of the local anesthetic so that he or she will not be concerned about the length of time it takes for the block to wear off. It is also essential that patients be instructed regarding protection of the extremity until sensation has completely returned. Finally, patients should be instructed to take their prescribed oral analgesics at the earliest sign of pain to mitigate against the analgesic gap that may otherwise develop. Additional methods to improve and or prolong postoperative analgesia include insertion of a brachial plexus catheter to provide continuous regional analgesia [51, 60] as well as the use of multimodal analgesia. In the multimodal approach use of a long-acting peripheral nerve block in combination with acetaminophen, NSAIDs (when not contraindicated), and oral opioid analgesics will minimize the total opioid requirements and their resulting side effects [61, 62].

## 12. Conclusion

The various approaches to the brachial plexus afford the anesthesiologist the ability to provide both excellent intraoperative anesthesia as well as postoperative analgesia with minimal complications and increased patient satisfaction following upper extremity surgery. The advantages over general anesthesia are numerous when performed in skilled hands. Ultrasound guidance with real-time needle visualization in relation to anatomic structures and target nerves makes regional anesthesia safer and more successful. With ultrasound guidance in experienced hands, brachial plexus blockade can lead to decreased block performance and onset time, increased success rate and decreased rate of complications. These advantages result in increased operating room efficiency, as well as increased patient and surgeon satisfaction.
